# Effects of Berries Consumption on Cardiovascular Risk Factors: A Meta-analysis with Trial Sequential Analysis of Randomized Controlled Trials

**DOI:** 10.1038/srep23625

**Published:** 2016-03-23

**Authors:** Haohai Huang, Guangzhao Chen, Dan Liao, Yongkun Zhu, Xiaoyan Xue

**Affiliations:** 1 Department of Clinical Pharmacy, Dongguan Third People’s Hospital, Affiliated Dongguan Shilong People’s Hospital of Southern Medical University, Dongguan, Guangdong, 523326, China; 2 Department of Pharmacy, Guangdong Province Agricultural Reclamation Central Hospital, Zhanjiang, Guangdong, 524002, China; 3 Department of Gynaecology & Obstetrics, Dongguan Maternal & Child Health Hospital, Dongguan, Guangdong, 523112, China

## Abstract

The effects of berries consumption on cardiovascular disease (CVD) risk factors have not been systematically examined. Here, we aimed to conduct a meta-analysis with trial sequential analysis to estimate the effect of berries consumption on CVD risk factors. PubMed, Embase, and CENTRAL were searched for randomized controlled trials (RCTs) that regarding the effects of berries consumption in either healthy participants or patients with CVD. Twenty-two eligible RCTs representing 1,251 subjects were enrolled. The pooled result showed that berries consumption significantly lowered the low density lipoprotein (LDL)-cholesterol [weighted mean difference (WMD), −0.21 mmol/L; 95% confidence interval (CI), −0.34 to −0.07; *P* = 0.003], systolic blood pressure (SBP) (WMD, −2.72 mmHg; 95% CI, −5.32 to −0.12; *P* = 0.04), fasting glucose (WMD, −0.10 mmol/L; 95% CI, −0.17 to −0.03; *P* = 0.004), body mass index (BMI) (WMD, −0.36 kg/m^2^; 95% CI, −0.54 to −0.18, *P* < 0.00001), Hemoglobin A1c (HbA1c) (WMD, −0.20%; 95% CI, −0.39 to −0.01; *P* = 0.04) and tumor necrosis factor-α (TNF-α) (WMD, −0.99 ρg/mL; 95% CI, −1.96 to −0.02; *P* = 0.04). However, no significant changes were seen in other markers. The current evidence suggests that berries consumption might be utilized as a possible new effective and safe supplementary option to better prevent and control CVD in humans.

Cardiovascular disease (CVD) is one of the leading causes of morbidity and mortality globally, accounting for 17.5 million CVD-related deaths annually in the United States and other countries and is projected to rise to almost 23.6 million deaths annually by the year 2030^1^,^2^. Although multiple risk factors for progression of CVD, dyslipidemia, which results from one or more abnormalities of blood lipids metabolism, remains a major key factor for this pathology and leads to the development of atherosclerotic plaques^3^,^4^. Observational epidemiologic evidence suggests that risk of heart attack in subjects with hyperlipidemia is 3 times higher than those in general population with normal lipid status, whereas a 1% decrease in serum cholesterol is strongly associated with 3% reduction in CVD risk^3^,^5^. Moreover, hypertension is also considered to be another important risk factor for CVD, since 60% of strokes and half of ischemic heart disease cases are attributable to elevated blood pressure (BP)^6^,^7^. From a public health perspective, nutraceuticals or functional foods composition intervention is considered a first approach in treating and controlling CVD^8^. In recent decades, both dietary therapy and pharmacologic interventions were used in CVD patients to improve their lipid profiles or BP. Multiple approaches to diet therapy have appreciable beneficial roles in preventing CVD, such as consumption of sour tea^9^, spirulina^10^ and barley-derived soluble fiber^11^.

Berries are rich in a number of polyphenols, including procyanidins, quercetin, phenolic acids, and particularly anthocyanins^12^,^13^. Accumulating evidence has shown that ingestion of berries display a wide range of biological activities in lowering the risk of CVD, including anti-inflammatory, antihypertensive, hypoglycaemic, anticoagulant and the improvement of lipid metabolism disorders^13^,^14^. However, randomized controlled trials (RCTs) have yielded conflicting results regarding this topic, and to our knowledge, there has not been any quantitative attempt to summarize the precise effect of berries consumption on cardiovascular risk factors. With accumulating evidence, we therefore performed a comprehensive assessment of the literature and carry out a meta-analysis by examining the change in lipid concentrations and BP induced by berries consumption. Additionally, we qualitatively reviewed other CVD risk factors that have been investigated in relation to berries consumption. Finally, we further applied trial sequential analysis (TSA) to determine whether the currently available evidence was sufficient and conclusive.

## Results

### Trial selection and trials characteristics

Based on the search strategy, the initial screening yielded 1,322 potentially eligible articles of which 45 were retrieved for complete review. The work of VALENTOVAÄ *et al*. was separated into 2 trials (since the intervention groups used different doses of cranberry juice on plasma lipid profiles)^15^. It should be noted that two trials conducted by Zhu *et al*. were reported in the same population^16^,^17^; therefore, we combined the informative data and retained only the latest article to avoid duplication of information^16^. Finally, 22 RCTs met our inclusion criteria and were included in the analysis of which one study was determined through checking reference lists of retrieved articles^15^,^16^,^18^,^19^,^20^,^21^,^22^,^23^,^24^,^25^,^26^,^27^,^28^,^29^,^30^,^31^,^32^,^33^,^34^,^35^,^36^. The flow diagram shows the process of literature screening, study selection, and reasons for exclusion, can be found as Supplemental Fig. S1.

The characteristics of treatment groups selected for analysis in the 22 RCTs are outlined in Table 1. Main characteristics are as follow: (1) Publishing year; these included studies were published from 2004 to 2015. (2) Intervention duration; 15 RCTs have treatment durations of 8 weeks or shorter^15^,^18^,^19^,^22^,^25^,^26^,^27^,^28^,^29^,^30^,^31^,^32^,^33^,^34^, and the remaining 7 RCTs have treatment durations more than 8 weeks^16^,^20^,^21^,^23^,^35^,^36^. (3) Number of patients; the trials varied in size from 18 to 146 subjects. A total of 1,251 subjects were included in these 22 RCTs. (4) Age of patients; the mean age of participants in each trial ranged from 21.5 to 65.5 years, with healthy status or risk of cardiovascular. (5) Types of berry; Of the 22 trials, 9 trials were used cranberry^15^,^18^,^20^,^21^,^27^,^28^,^29^,^32^, 5 trials were used bilberry^16^,^19^,^22^,^23^,^35^, 3 trials were used blueberry^25^,^26^,^30^, 2 trials were used whortleberry^33^,^34^, and 2 trials were used elderberry^24^,^31^, and 1 trial was used raspberry^36^. (6) Study design; most of the trials (20 trials) adopted parallel study designs^15^,^16^,^18^,^19^,^20^,^21^,^22^,^23^,^24^,^25^,^26^,^27^,^29^,^31^,^32^,^33^,^34^,^35^,^36^, and 2 trials used crossover designs^28^,^30^.

### Risk of Bias Assessment

Details of risk-of-bias analysis can be found as Supplemental Fig. S2. Overall, all satisfied the criteria of complete outcome data, selective reporting and other bias. All included trials were described as “random”. Adequate randomized sequence was generated in eight trials, but 12 studies lacked appropriately described randomization procedures^15^,^18^,^19^,^20^,^22^,^25^,^26^,^27^,^28^,^32^,^35^,^36^. Three trials did not mention whether the blind method was adopted or not, and were considered as at high risk of bias^18^,^19^,^20^.

### Primary outcome: Effect of berries consumption on lipid concentrations

**Figure 1:**
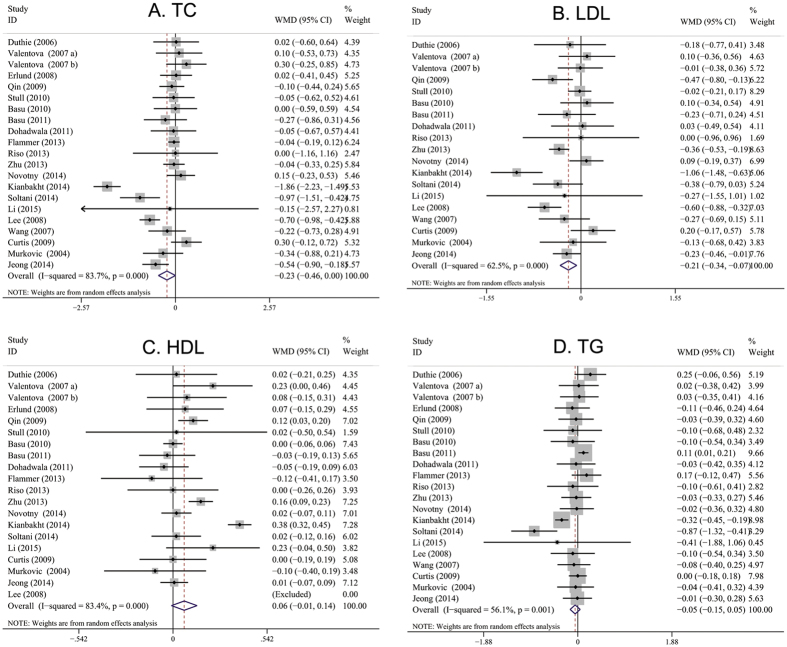
Meta-analysis of effects of berries products consumption on lipid parameters (A, TC; B, LDL; C, HDL; D, TG) compared with control arms. Sizes of data markers indicate the weight of each study in the analysis. WMD, weighted mean difference (the results were obtained from a random-effects model).

**Figure 2:**
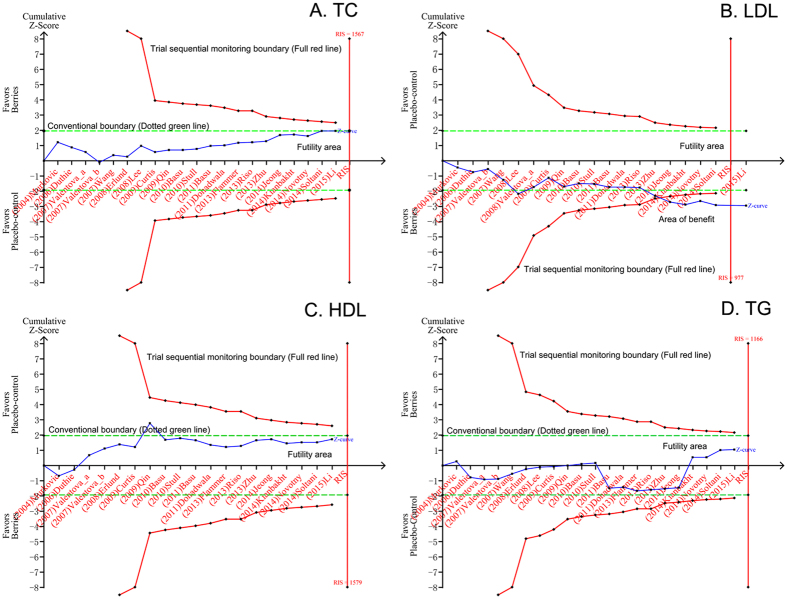
TSA on pooled result of effects of berries consumption on lipid profiles. **(A**) TSA on pooled result of TC: the cumulative sample size over the RIS of 1,606 and the cumulative Z-curve did not cross both the conventional boundary and the trial sequential monitoring boundary. **(B**) TSA on pooled result of LDL cholesterol: the cumulative sample size over the RIS of 1,082 and the cumulative Z-curve crossed both the conventional boundary and the trial sequential monitoring boundary for benefit. (**C**) TSA on pooled result of HDL cholesterol: the cumulative sample size over the RIS of 1,792 and the cumulative Z-curve did not cross both the conventional boundary and the trial sequential monitoring boundary. (**D**) TSA on pooled result of TG: the cumulative sample size over the RIS of 1,192 and the cumulative Z-curve did not cross both the conventional boundary and the trial sequential monitoring boundary. RIS, required information size.

The results for total cholesterol (TC) were reported in 21 studies that represented 1,133 participants^15^,^16^,^18^,^20^,^21^,^22^,^23^,^24^,^25^,^26^,^27^,^28^,^29^,^30^,^31^,^32^,^33^,^34^,^35^,^36^. Compared with placebo-control group, berries consumption did not significantly change the serum TC level [weighted mean difference (WMD), −0.24 mmol/L; 95% confidence interval (CI), −0.49 to 0.01; *P* = 0.06; Fig. 1A]. Heterogeneity was observed for this outcome (I^2^ = 83.7%). We undertook a TSA at the level of α of 0.05, β of 0.2, and then the required information size (RIS) of 1,567 was calculated. Z-curve does not cross the trial sequential monitoring boundary and the RIS has not been reached, which demonstrate that evidence to reach a conclusion is insufficient and further trials are warranted (Fig. 2A).

The mean change in low density lipoprotein (LDL)-cholesterol concentrations was reported in 19 studies representing 993 participants^15^,^16^,^18^,^20^,^21^,^23^,^24^,^25^,^26^,^27^,^28^,^30^,^31^,^32^,^33^,^34^,^35^,^36^. LDL-cholesterol was significantly lower in the berries-consumed subjects than in the placebo-treated subjects. The WMD in mean LDL cholesterol decreased by 0.21 mmol/L (95% CI: −0.34 to −0.07; *P* = 0.003; Fig. 1B), with significant heterogeneity (I^2^ = 62.5%). TSA was taken in the condition of α of 0.05, β of 0.2, and figured out RIS of 977. The accrued number of patients reached RIS, and the cumulative Z-curve cross conventional significance test boundary and RIS-adjusted boundary value, which established sufficient and conclusive evidence (Fig. 2B). Thus, further trials were not required and were unlikely to alter this conclusion.

The effect of berries on high density lipoprotein (HDL)-cholesterol was assessed in 20 trials based on the results of the meta-analysis^15^,^16^,^18^,^21^,^22^,^23^,^24^,^25^,^26^,^27^,^28^,^29^,^30^,^31^,^32^,^33^,^34^,^35^,^36^. The pooled result show that the berries intervention group may not significantly change in serum HDL-cholesterol level compared with that of controls (WMD, 0.06 mmol/L; 95% CI, −0.01 to 0.14; *P* = 0.08; Fig. 1C), with significant heterogeneity (I^2^ = 83%). TSA was performed and demonstrated RIS of 1,579. Z-curve does not cross any of the boundaries and the RIS has not been reached, which demonstrate that evidence to reach a conclusion is insufficient and further trials are warranted (Fig. 2C).

Twenty-one trials totaling 1,133 patients provided data on triglycerides (TG) level^15^,^16^,^18^,^20^,^21^,^22^,^23^,^24^,^25^,^26^,^27^,^28^,^29^,^30^,^31^,^32^,^33^,^34^,^35^,^36^. Figure 1D shows the pooled results from the random-effects model combing the WMD for the effect of berries consumption on TG level in the study population, which demonstrated that the level of TG was not significantly changed in the berries treatment group compared with the control group (WMD, −0.05; 95%CI, −0.15 to 0.05; *P* = 0.30), with significant heterogeneity among the studies (I^2^ = 56%). TSA was performed and RIS of 1,166 was counted. Z-curve does not cross any of the boundaries and the RIS has not been reached, which demonstrate that evidence to reach a conclusion is insufficient and more trials are needed (Fig. 2D).

### Secondary outcome: Effect of berries consumption on BP

**Figure 3:**
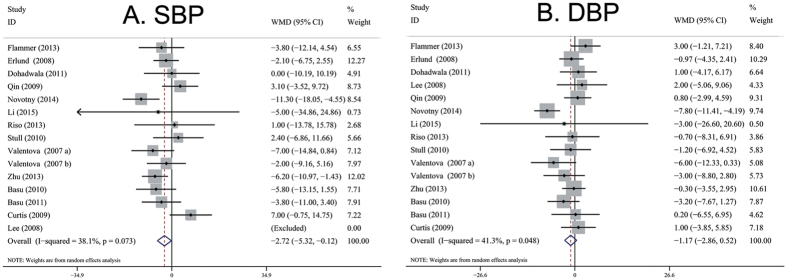
Meta-analysis of effects of berries consumption on BP (A, SBP; B, DBP) compared with control arms. Sizes of data markers indicate the weight of each study in the analysis. WMD, weighted mean difference (the results were obtained from a random-effects model).

Pooled results from the random-effects model showed that berries consumption significantly reduced the systolic blood pressure (SBP) level (15 trials with 832 individuals; WMD, −2.72 mmHg; 95% CI, −5.32 to −0.12; *P* = 0.04; I^2^ = 38%; Fig. 3A). No effect was found for diastolic blood pressure (DBP) (15 trials with 832 individuals; WMD, −1.17 mmHg; 95% CI, −2.86 to 0.52; *P* = 0.18; I^2^ = 41%; Fig. 3B). TSA was performed and the results can be found as Supplemental Fig. S3. TSA demonstrate that evidence to reach a conclusion is insufficient and more trials are needed, because of both of the Z-curves does not cross any of the boundaries and the RIS has not been reached.

### Other outcomes: Effect of berries consumption on other markers of cardiovascular disease

Other identified risk factors, such as markers of oxidative stress, markers of inflammation and endothelial function, were mentioned in our systematic literature search of clinical trials of berries consumption. Table 2 outline secondary outcomes. Compared with the control arms, berries consumption were associated with decreases in fasting glucose (WMD, −0.10 mmol/L; 95% CI, −0.17 to −0.03; *P* = 0.004), Hemoglobin A1c (HbA1c) (WMD, −0.20%; 95% CI, −0.39 to −0.01; *P* = 0.04), body mass index (BMI) (WMD, −0.36 kg/m^2^; 95% CI, −0.54 to −0.18, *P* < 0.00001) and tumor necrosis factor-α (TNF-α) (WMD, −0.99 ρg/mL; 95% CI, −1.96 to −0.02; *P* = 0.04), whereas no meaningful differences were observed for all other markers considered.

### Subgroup analyses and Sensitivity Analyses

Subgroup analyses were planned a priori to determine whether the mean age, intervention duration, types of berry, study design and type of patients modified the effects of berries on lipid profile. Briefly, based on the current subgroup analyses, significant reductions in the level of serum LDL cholesterol and significant increases the HDL-cholesterol level were observed in subjects with longer-term intervention duration (≥8 weeks), and bilberry consumption group. Statistical differences were also found in comparisons of whortleberry versus placebo-control in TC, LDL, and TG levels. A significant reduction was also observed in the level of serum TC and LDL cholesterol in subjects with black raspberry intervention. In a stratified analysis by type of patient, a significant reduction in TC and LDL concentration were observed in subjects with cardiovascular risk factors. Detailed subgroup results of lipids changes are summarized in Table 3.

In sensitivity analyses, the pooled effects of berries on lipid profile did not change after systematically dropping each trial. Furthermore, we also omitted the studies with high risk of bias. The aggregated results were similar when compared with the overall analysis (Table 3). All results of the sensitivity analysis suggest that the data in this meta-analysis are relatively stable and credible.

### Publication Bias

**Figure 4:**
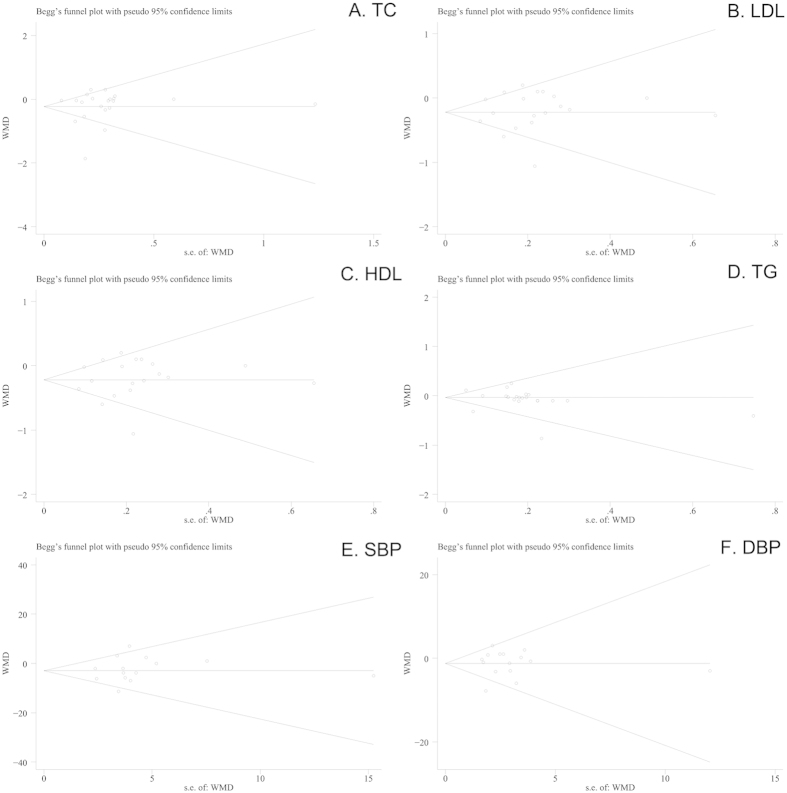
Tests for publication bias of effects of berries consumption on lipid profiles (A, TC; B, LDL; C, HDL, D, TG) and BP (E, SBP; F, DBP).

For the meta-analysis of the effect of berries consumption on lipid concentrations or BP, there was no evidence of publication bias by inspection of the funnel plot and formal statistical (for TC, Egger’s test *P* = 0.740; for LDL, Egger’s test *P* = 0.529; for HDL, Egger’s test *P* = 0.834; for TG, Egger’s test *P* = 0.815; for SBP, Egger’s test *P* = 0.511; for DBP, Egger’s test *P* = 0.488; respectively). No evidence of publication bias was indicated by the funnel plots (Fig. 4).

## Discussion

To our knowledge, this is the first meta-analysis to estimate of the relationship between berries consumption and changes of cardiovascular risk factors in subjects. Our analyses, with a total of 1,251 participants, showed a statistically significant reduction in LDL, SBP, fasting glucose, HbA1c, BMI and TNF-α levels after berries consumption. However, no statistically significant effects were observed in serum TC, TG, apolipoprotein (apo) A-I, apo B, HDL, DBP, oxidized LDL (ox-LDL), interleukin-6 (IL-6), C-reactive protein (CRP), soluble intercellular adhesion molecule-1 (sICAM-1) and soluble vascular cell adhesion molecule-1 (sVCAM-1) with berries ingestion when compared with placebo. The effects of berries on LDL-cholesterol reduction were found in several subgroups of individuals (i.e., longer-term intervention duration, and parallel study design). Similarly, subgroup analyses showed that significant reductions in the level of SBP were observed in subjects with shorter-term intervention duration (≤8 weeks), age ≥50 y, cranberry consumption group and parallel study design group. In addition, finding from subgroup analyzes also clearly show that berries products consumption significantly decreased the levels of serum TC, LDL-cholesterol and SBP in subjects with cardiovascular risk factors.

The precise mechanisms responsible for the presumed lipids-lowering properties of berries are not fully explored. However, an inhibition of cholesteryl ester transfer protein and the suppression of LDL oxidation, as well as improvement in HDL-associated paraoxonase 1 activity are considered the likely pathway^17^,^23^,^37^. Other mechanisms such as the inhibition of release reactive oxygen species from activated human granulocytes or suppress free-radical mediated lipid peroxidation and cell death in cultured aortic endothelial cells may also contribute to the antihyperlipidemic effect^38^. Furthermore, berries derived anthocyanins also showed similar beneficial properties *in vitro* by the promotion of cholesterol efflux from macrophages, which may also contribute to their beneficial effects on lipid profiles^39^. Similarly, the observed effects of berries intake on SBP is also supported by several *in vitro* mechanistic study findings, in which purified berries-derived anthocyanins have been shown to affect signaling pathways involved in inflammation and exert antiangiogenic effects including the inhibition of platelet-derived growth-factor signaling^40^. In an apolipoprotein E–deficient mice model, purified berries-derived anthocyanin ingestion directly inhibited atherosclerosis development and suppressed the development of atherosclerotic lesions^41^,^42^.

Treatment particularly aimed at decreasing LDL-cholesterol is effective in reducing the risk of death from coronary heart disease and stroke. Epidemiological and prospective studies have established the benefit of reducing LDL-C, with a 1% reduction associated with a 1% decrease in CVD events^43^. According to the updated clinical guidelines for cholesterol testing and management of the National Cholesterol Education Program (NCEP), the first priority of drug therapy have recommended progressively lower LDL-C for cholesterol treatment and CVD prevention as the primary target of therapy^43^. For this reason, treatment with LDL-lowering agents should be started as early as possible. Despite dramatic reductions in cardiovascular risk with intensively LDL-cholesterol lowering therapy, a substantial residual cardiovascular risk (up to 70% of baseline) remains significant after LDL-C goals are achieved with lipid-lowering treatments in most patient populations^44^,^45^. Early epidemiological studies have identified low levels of HDL-cholesterol (<1.0 mmol/L or 40 mg/dl) to be an independent determinant of increased cardiovascular risk^46^. Therefore, raising HDL cholesterol represents an important strategy for reducing residual cardiovascular risk in patients already optimally treated with LDL-cholesterol lowering therapy, and should lead to further improvements in clinical outcomes in these patient groups^47^. Analysis of the epidemiological data available suggests that cardiovascular risk increases by 2–3% for every increase of 0.03 mmol/L or 1 mg/dl HDL-C^48^. Dietary or dietary nutraceuticals composition intervention is now considered as potential candidates to protect against CVD for many reasons, including the poor adherence to drugs, contraindications to drugs, drug-related adverse effects (AEs) or personal preference for natural or alternative therapies. In our subgroup meta-analysis, the pooled result revealed that daily bilberries products consumption may significantly reduce the LDL-cholesterol level (0.38 mmol/L) and significant increases the HDL cholesterol level (0.14 mmol/L) without obviously AEs, which suggests that berries may be considered as a possible new effective and safe supplementary option for treatment of dyslipidemia and reduction of residual vascular risk in public population. Furthermore, this beneficial effect can be also found in subjects with longer-term intervention duration (≥8 weeks).

We also assessed the effects of berries products consumption on the other markers of cardiovascular disease. A number of studies have also examined the role of various proinflammatory cytokines in the progression of atherosclerosis^49^,^50^,^51^. CRP, a plasma protein synthesized by the liver, binds to LDL and is present in atherosclerotic plaques, so it has been proposed that CRP may have a causal role in coronary heart disease. VCAM-1, ICAM-1, IL-6, and TNF-α may provide additional information for cardiovascular risk stratification and prediction and have been regarded as useful biomarkers in assessing cardiovascular events in populations with various disease settings. In our present study, changes in plasma levels of CRP, IL-6, sVCAM-1, sICAM-1 and TNF-α did not differ between the berries and control groups. Furthermore, previous studies have demonstrated that the development of atherosclerosis is also accompanied by an accumulation of oxLDL^52^. In the present study, berries consumption, compared with control arms, produced a slight, but significant reduction of 0.99 ρg/mL (*P* = 0.04) in TNF-α level, whereas no meaningful differences were observed for all other antioxidant and inflammatory markers considered.

A major strength of this meta-analysis was following the PRISMA guidelines and the recommendations of the Cochrane Collaboration. To increase the robustness of our study, we applied TSA to assess the impact of random error and repetitive testing. Furthermore, funnel plot did not reflect obvious asymmetry, and Egger’s test further indicated no considerable publication bias in our present meta-analysis. This made our results more reliable to some extent. However, some potential limitations in the current study should be worthy of comment. Firstly, because of the various confounders appear in our screening trials, the lipid-lowering effect of berries consumption in subjects could be attributed to other healthy habits, such as cocoa, red wine, and tea consumption. The synergistic effects of other coexisting substances in berries foods on the clinical outcomes need to be excluded. Secondly, although extensive searches and clear inclusion criteria were made, it cannot be entirely sure that all relevant articles were selected, for the reason of the measures for lipid profiles or BP control were not primary outcomes in most of the trials selected in this meta-analysis, and the null findings of secondary outcomes may not have always been published. Finally, it should be noted that lipid profiles and BP change soon after switching diets, berries products consumption would need to be maintained indefinitely to maintain lower concentrations. Long-term compliance with consumption is often a concern with dietary interventions.

In conclusion, our meta-analysis showed that berries consumption significantly reduced the levels of LDL cholesterol, SBP, fasting glucose, HbA1c, BMI and TNF-α. However, no significantly changes were found in TC, HDL-cholesterol, TG, DBP, apo A-I, apo B, ox-LDL, IL-6, CRP, sICAM-1 and sVCAM-1. Our subgroup analyses demonstrated that berries products might be utilized as a possible new effective and safe supplementary option to better prevent and control CVD in subjects with cardiovascular risk factors. Further studies evaluating the effects of berries food consumption on clinical endpoints, such as cardiovascular morbidity and all-cause mortality, are required.

## Methods

### Literature search strategy

For this meta-analysis, we performed a search of PubMed, EMBASE, and the Cochrane Central Register of Controlled Trials (CENTRAL) databases from their inception to August 2015 to identify RCTs that examined the effects of berries consumption in either healthy participants or patients with cardiovascular diseases. A search strategy was performed with the use of exploded Medical Subject Heading (MeSH) terms and corresponding key words. Briefly, we used the following format of search terms: (“cranberry*” OR “raspberry*” OR “blueberr*” OR “whortleberry*” OR “bilberr*” OR “lingonberr*” OR “berr*” OR “Huckleberr*” OR “*Vaccinium macrocarpon* Ait” OR “*Vaccinium corymbosum* L.”). The search was limited to the criteria “clinical trials” and “human”, with no language restrictions. In order to identify RCTs, a highly sensitive filter was used. Two investigators independently screened the titles and abstracts resulting from the search strategies. Articles were excluded on initial screening if titles or abstracts were clearly irrelevant. Citation lists of retrieved articles were manually screened to ensure sensitivity of the search strategy. Bibliographies of accepted studies and recent reviews were also screened to ensure a complete study listing.

### Study selection criteria

To be selected for analysis, full publications were retrieved for evaluation on the basis of criteria established a priori. Studies were selected for this analysis if they met the following criteria: *1*) subjects needed to have specifically ingested the berries interventions; *2*) study was an RCT in human with either a parallel or crossover design; *3*) studies used placebo as comparison intervention; *4*) food-intake control regimens of experimental groups were consistent with those of control groups; *5*) studies had to assess at least one primary outcomes [i.e., lipids (TC, LDL, HDL, TG), BP (SBP, DBP) or other pre-determined CVD risk factors]; *6*) the baseline and endpoint information (or their difference) with SDs, SEMs, or 95% CIs were available for each group in the study.

### Risk of bias assessment

Risk-of-bias assessment was performed in accordance with guidelines outlined in the cochrane risk-of-bias tool^53^. For each study, two investigators subjectively assigned a value of ‘high’, ‘low’, or ‘unclear’ to the following 7 domains: random sequence generation; allocation concealment; blinding of participants and personnel; blinding of outcome assessment; incomplete outcome data; selective reporting; and other bias.

### Data extraction

Two investigators independently collected the data, crosschecked and reached a consensus on all items. The following variables were extracted from the including studies: first author’s name, publication year, number of subjects enrolled (intervention/control), types of study design, patients’ characteristics [including mean age, sex, BMI, health status, baseline LDL and TC levels, baseline SBP and DBP], medication strategies of treatment and control arms, duration of treatment, location, and outcome measures for the analysis. When the same patients were reported in several publications, we retained only the largest study to avoid duplication of information. If trials included in our analysis have multiple intervention groups, we grouped together all the experimental groups and compared them with the control group, respectively. Any disagreements in abstracted data between the reviewers were adjudicated by a third author.

### Data synthesis and statistical analysis

The primary end points of this study were the mean difference in lipid profiles (mmol/L; TC, LDL, HDL and TG). Secondary outcome was the mean difference in BP (mmHg; SBP and DBP). Net changes in each of the study variables, which were calculated from baseline and follow-up means and SDs, were used to estimate the principal effect. Intention to treat (ITT) data from all eligible trials was used in this meta-analysis. Since ITT is intended to avoid various misleading artifacts that can arise in intervention research such as non-random attrition of participants from the study or crossover^54^. Studies that reported results in mg/dL were converted to mmol/L using the standard conversion factors. The conversion factor was 1 mg/dL = 0.02586 mmol/L for cholesterol, 1 mg/dL = 0.01129 mmol/L for TG and 1mg/dL = 0.056 mmol/L for glucose. These values were captured as mean ± SD. When SDs were not directly available, they were calculated from SE or 95% CI according to the *Cochrane Handbook for Systematic Reviews of Interventions*^55^. Other cardiovascular risk factors were also captured in this way, including apo A-I and apo B, BMI, ox-LDL, glucose, HbA1c, IL-6, TNF-α, VCAM-1, ICAM-1 and CRP.

Our meta-analysis and statistical analyses were performed with STATA (version 12; StataCorp, College Station, TX). For continuous outcomes, summary estimates of WMD with 95% CI were calculated for net changes for each intervention and control group. Clinical heterogeneity was assessed by considering the design of each study. The statistical heterogeneity across studies was tested by using the I^2^ statistic, which was a quantitative measure of inconsistency across studies. We considered an I^2^ value 50% to indicate significant heterogeneity between the trials^56^. Primary analyses were done with a fixed effects model; secondary confirmatory analyses were done with a random effects model if there was significant heterogeneity. To check the influence of various factors on cardiovascular risk factors of berries intervention, we further performed a priori subgroup analysis according to participant characteristics. The subgroup analyses were performed only for the effect of berries products on lipid profiles and BP because of small numbers of studies for other outcome. Furthermore, sensitivity analysis was also performed by removing each study at a time to evaluate the stability of the results. Furthermore, we evaluated potential publication bias of the studies included with Funnel plots and Egger’s regression asymmetry test (*P* < 0.05 was considered representative of statistically significant publication bias)^57^. All tests were two-tailed and a *P* value of less than 0.05 was deemed statistically significant.

### Trial sequential analysis

The estimation of sample size is required by the repeatability principle of clinical trials aiming at the study power in statistics. In a meta-analysis, repeated significance test of sparse and accumulated data may increase the risk of random errors which cause false positive or negative results^58^,^59^. A method that aims to correct for the increased risk of random errors is called Trial sequential analyses, which were performed post hoc to assess the risk of random errors, false positive results, and to help clarify the need for additional trials. If the cumulative Z-curve crosses the trial sequential monitoring boundary or enters the futility area, then we can draw the conclusion that a sufficient level of evidence for the anticipated intervention effect may have been reached and no further trials are needed; whereas the Z-curve does not cross any of the boundaries and the required information size has not been reached, evidence to reach a conclusion is insufficient.

The primary outcomes in our meta-analysis were continuous data category, so we estimated the RIS based on the empirical data autogenerated from software according to the data input. The TSA was performed at the level of an overall 5% risk of a type I error (two sided) and 20% of the type II error (a statistical test power of 80%)^60^. TSA software (version 0.9 beta; http://www.ctu.dk/tsa) were used for these analyses.

## Additional Information

**How to cite this article**: Huang, H. *et al*. Effects of Berries Consumption on Cardiovascular Risk Factors: A Meta-analysis with Trial Sequential Analysis of Randomized Controlled Trials. *Sci. Rep*. **6**, 23625; doi: 10.1038/srep23625 (2016).

## Supplementary Material

Supplementary Information

## Figures and Tables

**Table 1:** Characteristics of the 22 included randomized controlled trials^1^.

Study	Year	No. of patients	Type of study	Type of Patient	Initial BMI	Initial |TC/LDL-c^2^	Mean age^2^	Gender (M/F)	Treatment group	Control group	Design duration	Initial SBP/DBP^2^	Location	Outcomes of interest
Murkovic *et al*.^31^	2004	34	R, DB, PC, P	Healthy	23.6	5.0/2.8	29.0	20/14	Elderberry juice	Placebo	2 week	NA/NA	Austria	TC, LDL, HDL, TG
Duthie *et al*.^18^	2006	20	R, PC, P	Healthy	NA	4.7/2.9	27.8	0/20	Cranberry juice	Placebo	2 week	NA/NA	UK	TC, LDL, HDL, TG
Valentova *et al*.^15^	2007a	27	R, DB, PC, P	Healthy	20.8	4.7/2.7	21.5	0/27	Cranberry juice	Placebo	8 week	114/75	Czech	TC, LDL, HDL, TG, BP
Valentova *et al*.^15^	2007b	30	R, DB, PC, P	Healthy	21.2	4.7/2.6	21.5	0/57	Cranberry juice	Placebo	8 week	112/73	Czech	TC, LDL, HDL, TG, BP
Karlsen *et al*.^19^	2007	118	P, PC, P	Healthy	24.5	NA/NA	61	NA	Bilberry, black currants	Placebo	3 weeks	NA/NA	Norway	IL-6, CRP, TNF-α,
Wang *et al*.^20^	2007	40	R, PC, P	Healthy	21.0	4.5/2.9	NA	20/20	Cranberry vinegar	Placebo	10 week	NA/NA	China	TC, LDL, TG
Lee *et al*.^21^	2008	30	R, DB, PC, P	Type 2 diabetes	26	5.2/3.1	65.5	16/14	Cranberry capsule	Placebo	12 week	130/69	China	TC, LDL, HDL, TG, BP, BMI, CRP, ox-LDL, glucose, HbA1c
Erlund *et al*.^22^	2008	71	R,SB, PC, P	Cardiovascular risk factors	26.2	6.3/NA	57.9	25/46	Bilberry, lingonberry	Placebo	8 week	129/81	Finland	TC, HDL, TG, BP, sICAM-1
Qin *et al*.^23^	2009	120	R, DB, PC, P	Dyslipidemic	26.1	5.8/4.1	40–65	42/78	Bilberry, blackcurrant	Placebo	12 week	128/83	China	TC, LDL, HDL, TG, BP, BMI, apo A-I, apo B, glucose
Curtis *et al*.^24^	2009	52	R, DB, PC, P	Healthy postmenopausal	24.7	5.5/3.5	58.2	0/52	Elderberry	Placebo	12 week	126/80	UK	TC, LDL, HDL, TG, BP, IL-6, BMI, CRP, TNF-α, glucose
Stull *et al*.^25^	2010	32	R, DB, PC, P	Obese, insulin-resistant	37.4	5.3/3.2	51.5	5/27	Blueberry smoothie	Placebo	6 week	120/75	USA	TC, LDL, HDL, TG, BP, BMI, CRP, TNF-α, glucose
Basu *et al*.^26^	2010	48	R, SB, PC, P	Obese, metabolic, syndrome	37.8	NA/NA	50	4/44	Blueberry beverage	Placebo	8 week	NA/NA	USA	TC, LDL, HDL, TG, BP, IL-6, CRP, sVCAM-1, sICAM-1, ox-LDL, glucose, HbA1C
Basu *et al*.^27^	2011	31	R, DB, PC, P	Metabolic syndrome	40.0	3.4/3.1	52.0	Na	Cranberry juice	Placebo	8 week	132/83	USA	TC, LDL, HDL, TG, BP, IL-6, CRP, ox-LDL, glucose
Dohadwala *et al*.^28^	2011	44	R, DB, PC, C	Coronary artery disease	29.5	4.1/2.3	62.0	30/14	Cranberry juice	Placebo	4 week	132/73	USA	TC, LDL, HDL, TG, BP, CRP, sICAM-1, glucose, HbA1c
Flammer *et al*.^29^	2013	69	R, DB, PC, P	Cardiovascular risk factors	27.4	4.7/NA	48.1	31/38	Cranberry juice	Placebo	8 week	116/71	USA	TC, HDL, TG, BP, IL-6, CRP, TNF-α, sVCAM-1, sICAM-1, ox-LDL
Riso *et al*.^30^	2013	18	R, PC, C	Cardiovascular risk factors	24.8	5.8/3.8	47.8	NA	Blueberry drink	Placebo	6 week	122/80	Italy	TC, LDL, HDL, TG, BP, IL-6, BMI, CRP, TNF-α, sVCAM-1, glucose
Zhu *et al*. 16	2013	146	R, DB, PC, P	Hypercholesterole-mia	26.6	6.5/3.3	40-65	NA	Bilberry, blackcurrant	Placebo	24 week	125/84	China	TC, LDL, HDL, TG, BP, apo A-I, apo B, CRP, TNF-α, sVCAM-1
Novotny *et al*.^32^	2014	56	R, DB, PC, P	Healthy	28.0	5.1/3.2	50.0	26/30	Cranberry Juice	Placebo	8 week	117/71	USA	TC, LDL, HDL, TG, BP, BMI, apo A-I, apo B, CRP, sVCAM-1, sICAM-1, glucose
Kianbakht *et al*.^33^	2014	80	R, DB, PC, P	Hyperlipidemia	29.9	7.6/4.3	53.5	38/42	Whortleberry	Placebo	8 week	NA/NA	Iran	TC, LDL, HDL, TG
Soltani *et al*.^34^	2014	50	R, DB, PC, P	Hyperlipidemia	25.3	5.8/3.3	47.2	20/30	Whortleberry	Placebo	4 week	NA/NA	Iran	TC, LDL, HDL, TG, BMI, CRP
Li *et al*.^35^	2015	58	R, DB, PC, P	Diabetic	24.0	5.0/3.2	57.8	34/24	Bilberry	Placebo	24 week	129/81	China	TC, LDL, HDL, TG, BP, IL-6, BMI, apo A-I, apo B, TNF-α, glucose, HbA1c
Jeong *et al*.^36^	2014	77	R, DB, PC, P	Metabolic syndrome	25.7	5.1/2.5	59.0	36/41	Black Raspberry	Placebo	12 week	NA/NA	Korea	TC, LDL, HDL, TG, apo A-I, apo B, IL-6, CRP, TNF-α,, sVCAM-1, sICAM-1

^1^R, randomized; DB, double-blind; SB, single-blinded; PC, placebo controlled; P, parallel; C, crossover; NA, not available; M, male; F, female; TC, total cholesterol; LDL, low density lipoprotein cholesterol; HDL, high density lipoprotein cholesterol; HbA1c, Hemoglobin A1c; TG, triglycerides; SBP, systolic blood pressure; DBP, diastolic blood pressure; sICAM, soluble intercellular adhesion molecule; sVCAM, soluble vascular cell adhesion molecule; CRP, C-reactive protein; TNF-α, Tumor Necrosis Factor-α; apo, apolipoprotein; ox-LDL, oxidized LDL; IL-6, interleukin-6.

^2^Values for age, BMI, baseline TC, LDL-c, SBP and DBP are means unless otherwise stated. For baseline TC and LDL-c, mmol/L; for baseline SBP and DBP, mm Hg; for BMI, kg/m^2^.

**Table 2:** Effect of berries consumption on other markers of cardiovascular disease^1^.

Markers outcomes	No. Trials	No. Patients	WMD (95% CI)^1^	*P* Value	*I*^*2*^, %	*P* Value of Heterogeneity	Model used
Glucose (mmol/L)	10	489	−0.10 (−0.17 to −0.03)*	0.004	0	0.62	F
HbA1c (%)	3	136	−0.20 (−0.39 to −0.01)*	0.04	80	0.006	R
BMI (kg/m^2^)	8	416	−0.36 (−0.54 to −0.18)*	0.0001	0	1.00	F
Apo A-I (mg/dL)	5	457	0.85 (−3.53 to 5.23)	0.70	50	0.09	R
Apo B (mg/dL)	5	457	−3.68 (−10.48 to 3.13)	0.29	75	0.003	R
Ox-LDL (μmol/l)	4	178	−3.45 (−10.01 to 3.11)	0.30	40	0.17	R
IL-6 (ρg/mL)	8	471	−0.14 (−0.45 to 0.16)	0.35	40	0.11	R
TNF-alpha (ρg/mL)	8	570	−0.99 (−1.96 to −0.02)*	0.04	21	0.26	R
CRP (mg/L)	13	771	−0.08 (−0.30 to 0.15)	0.52	0	0.98	F
sICAM-1 (ηg/mL)	6	365	3.61 (−9.85 to 17.08)	0.60	53	0.06	R
sVCAM-1 (ηg/mL)	6	414	−13.55 (−65.95 to 38.84)	0.61	75	0.001	R

^1^R, Random-effects model; F, Fixed-effects model; WMD, weighted mean difference; CI, confidence interval; *Indicates a significant result.

**Table 3:** Subgroup estimation of the effects of berries consumption on lipid concentrations according to predefined study characteristics^1^.

Variables	No. trials	Total cholesterol			No. trials	LDL cholesterol			No. trials	HDL cholesterol			No. trials	Triglycerides		
		WMD (95%CI)	*P*	I^2^(%)		WMD (95%CI)	*P*	I^2^(%)		WMD (95%CI)	*P*	I^2^(%)		WMD (95%CI)	*P*	I^2^(%)
**Total**	21	−0.23 (−0.46 to 0.00)	0.05	84	19	−0.21 (−0.34 to −0.07)*	0.003	62	20	0.06 (−0.01 to 0.14)	0.08	83	21	−0.05 (−0.15 to 0.05)	0.30	56
**Subgroup analysis**																
*Mean age*																
<50 y	8	−0.12 (−0.38 to 0.13)	0.34	49	7	−0.08 (−0.26 to 0.11)	0.41	0	8	0.01 (−0.04 to 0.06)	0.68	0	8	−0.05 (−0.27 to 0.17)	0.64	61
≥50 y	10	−0.34 (−0.80 to 0.12)	0.14	90	9	−0.22 (−0.47 to 0.02)	0.07	78	10	0.08 (−0.07 to 0.22)	0.30	90	10	−0.06 (−0.21 to 0.08)	0.38	67
*Intervention duration*																
shorter-term (≤8 weeks)	14	−0.23 (−0.57 to 0.11)	0.19	87	12	−0.14 (−0.32 to 0.05)	0.14	58	14	0.05 (−0.06 to 0.15)	0.38	87	14	−0.06 (−0.21 to 0.08)	0.40	71
longer-term (>8 weeks)	7	−0.23 (−0.52 to 0.07)	0.13	73	7	−0.31 (−0.48 to −0.13)*	0.0006	54	6	0.09 (0.02 to 0.17)*	0.02	61	7	−0.03 (−0.14 to 0.09)	0.63	0
*Types of berry*																
Cranberry juice	9	−0.11 (−0.34 to 0.13)	0.36	65	8	−0.15 (−0.36 to 0.06)	0.17	55	8	0.01 (−0.05 to 0.07)	0.67	0	9	0.09 (−0.00 to 0.17)	0.05	0
Bilberry	4	−0.05 (−0.24 to 0.15)	0.63	0	3	−0.38 (−0.53 to −0.23)*	0.0001	0	4	0.14 (0.09 to 0.19)*	0.0001	0	4	−0.06 (−0.25 to 0.13)	0.53	0
Blueberry	3	−0.02 (−0.41 to 0.36)	0.91	0	3	−0.00 (−0.17 to 0.17)	0.99	0	3	0.00 (−0.05 to 0.05)	0.99	0	3	−0.10 (−0.39 to 0.19)	0.50	0
Whortleberry	2	−1.44 (−2.32 to −0.56)*	0.001	86	2	−0.72 (−1.38 to −0.05)*	0.03	80	2	0.21 (−0.15 to 0.56)	0.26	95	2	−0.55 (−1.08 to −0.02)*	0.04	80
Black Raspberry	1	−0.54 (−0.90 to −0.18)*	0.003	NA	1	−0.24 (−0.46 to −0.01)*	0.04	NA	1	0.01 (−0.07 to 0.09)	0.86	NA	1	−0.01 (−0.30 to 0.28)	0.96	NA
Elderberry	2	−0.01 (−0.61 to 0.63)	0.98	70	2	0.10 (−0.21 to 0.40)	0.53	0	2	−0.03 (−0.19 to 0.13)	0.71	0	5	−0.01 (−0.17 to 0.15)	0.92	0
*Type of Patient*																
Healthy	7	0.08 (−0.11 to 0.26)	0.43	0	7	0.01 (−0.14 to 0.16)	0.88	0	6	0.03 (−0.03 to 0.10)	0.31	0	7	0.02 (−0.09 to 0.13)	0.72	0
Cardiovascular risk factors	14	−0.37 (−0.68 to −0.06)*	0.02	88	12	−0.31 (−0.49 to −0.14)*	0.0003	66	14	0.07 (−0.02 to 0.17)	0.13	88	14	−0.10 (−0.25 to 0.04)	0.18	69
*Study design*																
Parallel	19	−0.24 (−0.49 to 0.00)	0.05	85	17	−0.22 (−0.36 to −0.08)*	0.003	66	18	0.08 (−0.00 to 0.15)	0.06	85	19	−0.05 (−0.16 to 0.05)	0.33	60
Crossover	2	−0.04 (−0.59 to 0.51)	0.88	0	2	0.02 (−0.43 to 0.47)	0.93	0	2	−0.04 (−0.16 to 0.08)	0.53	0	2	−0.06 (−0.37 to 0.25)	0.71	0
*Types of interventions*																
whole berries	17	−0.29 (−0.56 to −0.01)*	0.04	86	15	−0.20 (−0.36 to −0.04)*	0.02	63	16	0.05 (−0.05 to 0.14)	0.35	86	17	−0.06 (−0.18 to 0.06)	0.33	65
Purified berries-derived anthocyanins	4	0.02 (−0.18 to 0.21)	0.86	0	4	−0.24 (−0.54 to 0.07)	0.13	65	4	0.14 (0.08 to 0.19)*	0.0001	2	4	−0.02 (−0.15 to 0.12)	0.20	0
**Sensitivity analysis**																
Exclude high-risk research	19	−0.24 (−0.49 to 0.01)	0.06	85	17	−0.20 (−0.35 to −0.05)*	0.008	67	19	0.07 (−0.01 to 0.14)	0.09	84	19	−0.07 (−0.18 to 0.04)	0.20	57

^1^NA, Not applicable; *Indicates a significant result.
