# Supplementary material for: Effects of Berries Consumption on Cardiovascular Risk Factors: A Meta-analysis with Trial Sequential Analysis of Randomized Controlled Trials
Source: Sci Rep. 2016 Mar 23;6:23625. doi: 10.1038/srep23625 (PMC4804301; doi:10.1038/srep23625)

Effects of Berries Consumption on Cardiovascular Risk Factors: A Meta-analysis with Trial Sequential Analysis of Randomized Controlled Trials

Running Head: Berries Consumption on CVD Risk Factors

Haohai Huang[1,*], Guangzhao Chen[2], Dan Liao[3], Yongkun Zhu[1] & Xiaoyan Xue[1]

Authors' Affiliations:

[1]Department of Clinical Pharmacy, Dongguan Third People's Hospital, Affiliated Dongguan Shilong People's Hospital of Southern Medical University, Dongguan, Guangdong, 523326, China

[2]Department of Pharmacy, Guangdong Province Agricultural Reclamation Central Hospital, Zhanjiang, Guangdong, 524002, China

[3]Department of Gynaecology & Obstetrics, Dongguan Maternal & Child Health Hospital, Dongguan, Guangdong, 523112, China

***Address correspondence to:** Haohai Huang, M.D. Department of Clinical Pharmacy, Third People's Hospital of Dongguan, Affiliated Dongguan Shilong People's Hospital of Southern Medical University, No.1, Huangzhou Xianglong Road of Shilong Town, Dongguan, Guangdong, 523326, China. **Fax:** 86 0769 81368123; **E-mail address:** haohaihuang@hotmal.com (H. Huang).

Conflict of interest:

Haohai Huang, Guangzhao Chen, Dan Liao, Yongkun Zhu and Xiaoyan Xue have no conflict of interest related to this work. All authors reviewed and approved the final article.

Supplemental Information

Supplemental Figure S1. Flowchart of database searches and articles included in the present meta-analysis.

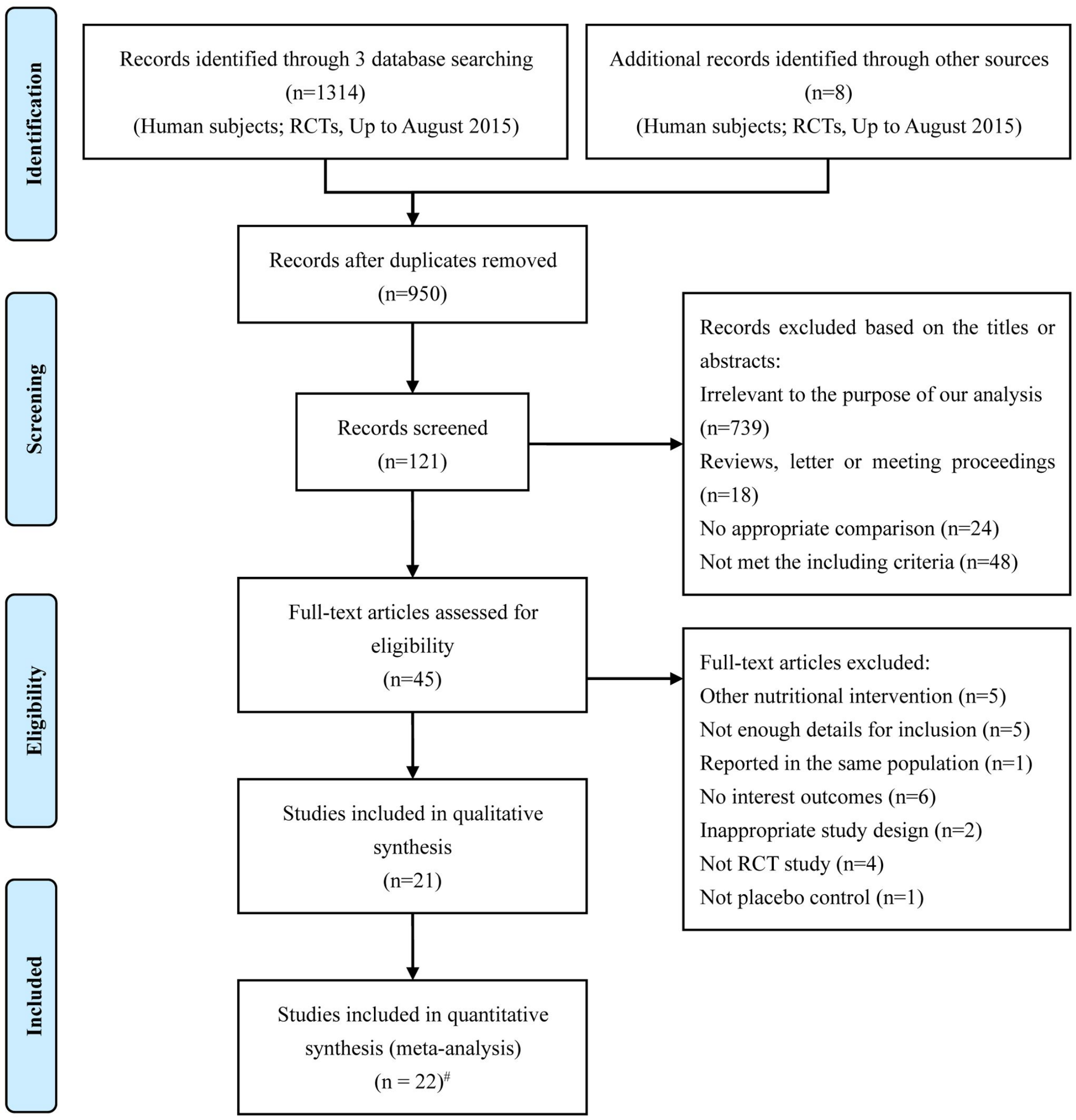

Pubmed, http://www.ncbi.nlm.nih.gov/pubmed/;

Embase, http://www.embase.com/home;

CochraneLibrary, http://www.thecochranelibrary.com/view/0/index.html;

#The work conducted by VALENTOVAÄ et al. was separated into 2 trials.

Supplemental Figure S2. Results of risk of bias assessment **A**: Risk of bias graph: review authors' judgments about each risk of bias item presented as percentages across all included studies. **B**: Risk of bias summary: review authors' judgments about each risk of bias item for each included study.

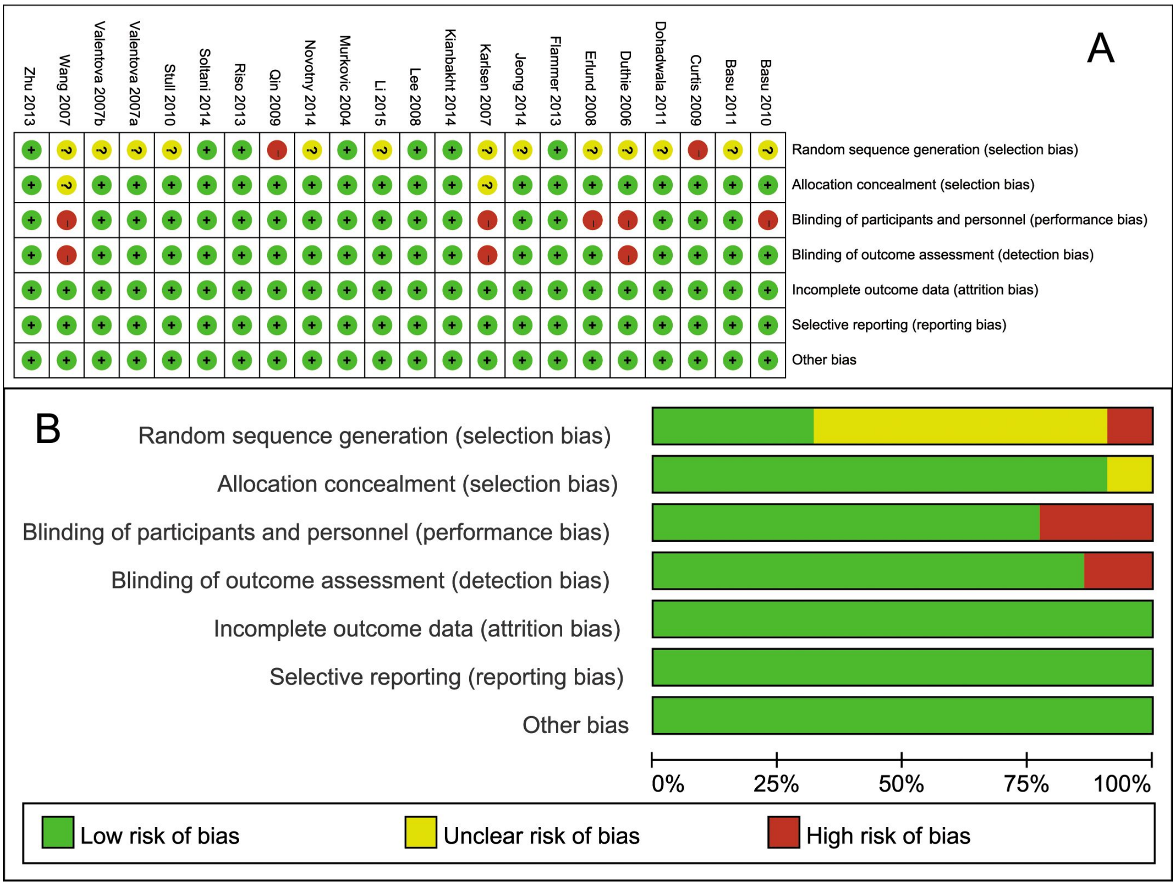

Supplemental Figure S3. TSA on pooled result of effects of berries consumption on BP. A, TSA on pooled result of SBP: the cumulative sample size over the RIS of 1,392 and the cumulative Z-curve crossed the conventional boundary but did not cross the trial sequential monitoring boundary. **B**, TSA on pooled result of DBP: the cumulative sample size over the RIS of 2,016 and the cumulative Z-curve did not cross both the conventional boundary and the trial sequential monitoring boundary for benefit. RIS, required information size

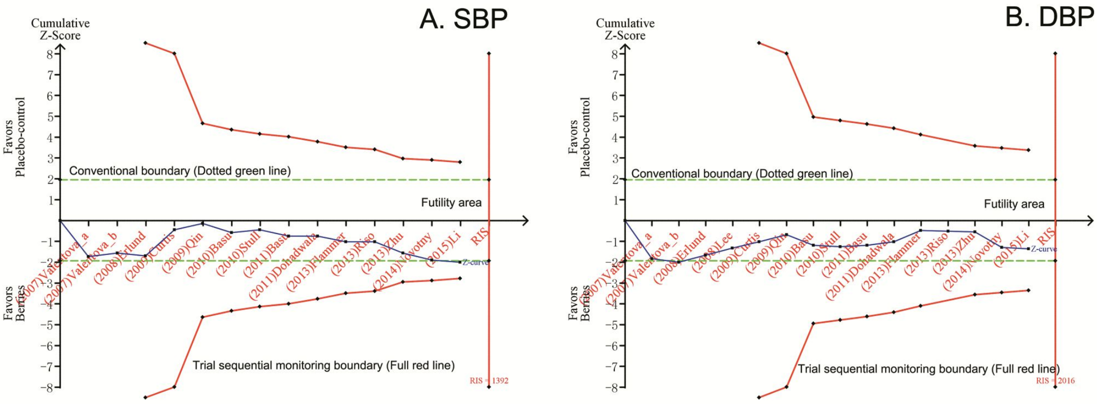

Supplement: Supplementary Information [file srep23625-s1.pdf]
